# The spiritual history in outpatient practice: attitudes and practices of health professionals in the Adventist Health System

**DOI:** 10.1186/s12909-017-0938-8

**Published:** 2017-06-12

**Authors:** Harold G. Koenig, Kathleen Perno, Ted Hamilton

**Affiliations:** 1 Department of Psychiatry, Duke University Medical Center, Box 3400, Durham, NC 27710 USA; 2 Department of Medicine, King Abdulaziz University, Jeddah, Saudi Arabia; 3 School of Public Health, Ningxia Medical University, Yinchuan, People’s Republic of China; 4 Medical Mission Integration, Adventist Health System, Orlando, FL USA

**Keywords:** Physician attitudes, Physician behavior, Spirituality, Religion, Spiritual history

## Abstract

**Background:**

A screening spiritual history (SSH) is how health professionals (HP) identify patients’ spiritual values, beliefs and preferences (VBPs) in the outpatient setting. We report on attitudes and practices of HPs in the largest Protestant health system in the U.S., the Adventist Health System (AHS).

**Method:**

Physicians or mid-level practitioners (*N* = 1082) in AHS-affiliated practices were approached and 513 (47%) agreed to participate. Participants were asked to identify a “spiritual care coordinator” (nurse/staff) and complete a questionnaire that assessed demographics, practice characteristics, religious involvement, and attitudes/practices concerning the SSH. Prevalence and predictors of attitudes/practices were identified.

**Results:**

Questionnaires were completed by 427 physicians, 86 mid-level practitioners, and 224 nurses/staff (i.e., spiritual care coordinators). Among physicians, 45% agreed that HPs should take a SSH; of mid-level practitioners, 56% agreed; and of nurses/staff, 54% agreed. A significant proportion (range 31–54%) agreed that physicians should take the SSH. Participants indicated a SSH is appropriate for all outpatients (46–57%), well-visit exams (50–60%), the chronically ill (71–75%) and terminally ill (79–82%). A majority agreed the SSH should be documented in the medical record (67–80%). Few (11–17%) currently took a SSH, although most were at least sometimes willing to take a SSH (87–94%) or review the results thereof (86–98%). Self-rated importance of religion was the strongest predictor of SSH attitudes/practices.

**Conclusions:**

Many in the AHS say a SSH should be done, are willing to do it, and are willing to review the results, although few currently do so. Education, training, and support may help HPs identify and address patients' spiritual VBPs.

## Background

Adventist Health System (AHS) with 46 hospital campuses staffed by 77,000 employees in 10 states sees 4.7 million patients annually [1], is the 5th largest non-profit hospital system [2], and is the largest Protestant health care system in the United States. The stated mission of AHS is “Extending the healing ministry of Christ.” How exactly AHS carries out this mission while maintaining its religious identity in a pluralistic society with both health professionals and patients from a wide variety of religious and non-religious backgrounds, and meeting the requirements necessary to receive Medicare and Medicaid, has challenged AHS leadership -- as it has in other faith-based health systems (FBHSs) [3]. Compared to investor-owned or government systems, there is evidence that FBHSs have better overall quality performance, lower risk-adjusted mortality, decreased length of stay, and higher Hospital Consumer Assessment of Healthcare Providers and Systems (HCAHPS) scores [4, 5]. FBHSs may be more likely identify and address patients’ spiritual needs.

Although there is little agreement in the field of religion, spirituality and health more generally on exactly what the word “spiritual” means, considerable progress has been made in the nursing and palliative care literature [6–8], and an accepted definition (arrived at by consensus) has been arrived at in the area of palliative care [9] that has been used in prior studies [10, 11]. However, as used in the present context (general outpatient medical care), we define spirituality as follows: “Spirituality is distinguished from all other things—humanism, values, morals, and mental health—by its connection to that which is sacred, the transcendent. The transcendent is that which is outside of the self, and yet also within the self — and in Western traditions is called God, Allah, HaShem or a Higher Power, and in Eastern traditions may be called Brahman, manifestations of Brahman, Buddha, Tao or Ultimate Truth/Reality. Spirituality is intimately connected to the supernatural, the mystical, and to organized religion, although it also extends beyond organized religion (and begins before it). Spirituality includes both the search for the transcendent and the discovery of the transcendent, and so involves traveling along the path that leads from non-consideration to questioning to either stanch nonbelief or belief, and if belief, then ultimately to devotion, and finally, to surrender” (p 46) [12]. Thus, as used here, spiritual includes religion. However, the scope of spiritual assessment is quite broad and patient-centered in clinical practice.

Few health professionals (HPs) regularly take a screening spiritual history (SSH) to identify patients’ spiritual beliefs, values or needs. This is true despite growing research linking religion/spirituality (R/S) to health [12], use of R/S to cope with illness [13], and widespread spiritual needs reported by patients [14–16]. It is true despite the influence of R/S beliefs on medical decision making [17–20], requirements by accrediting organizations to respect patients’ personal values, beliefs, and preferences [21], encouragement by the American Association of Medical Colleges to address spiritual issues [22], and the receptiveness of patients [23].

A survey of 1144 U.S. physicians (latest national data available) found that only 10% said they often inquired about R/S issues, confirming earlier reports from regional surveys [24–26]. Infrequent assessment by physicians is likewise documented in more recent surveys, indicating that 9–63% (median 34%) report often or always taking a R/S history, with higher figures for end-of-life, inpatient, and psychiatric settings [27–31]. Failure to assess and address patients’ spiritual needs is associated with poorer quality of life [32], reduced satisfaction with care [33], and increased costs during the last week of life [34, 35].

Taking a SSH can itself have benefits. For example, researchers conducted a clinical trial to assess short-term outcomes from oncologists taking a SSH in 118 cancer outpatients [36]. At the 3-week follow-up, the intervention group had significantly fewer depressive symptoms, increased sense of interpersonal caring by the physician, and higher functional well-being.

Primary barriers to HPs assessing and addressing patients’ spiritual needs have been identified. Failure to do so is primarily because of (1) HP discomfort with spirituality and (2) lack of training [37, 38]. Consequently, training to assess and address patients’ spiritual needs has been recommended as “the critical next step” to meet national care quality standards in healthcare [38].

### Objectives

Aims of the present study were to (1) identify attitudes and behaviors regarding the SSH in physician outpatient practices affiliated with the AHS, and (2) determine sociodemographic, professional, and religious predictors of those attitudes and behaviors.

## Methods

### Sample

Between February and August 2015, a total of 1082 physician or mid-level practitioners (MLPs, i.e., nurse practitioners or physicians assistants) affiliated with the AHS in Florida, Georgia, Illinois, and North Carolina (approximately 50% of AHS hospitals) were approached to participate in a 12-month project that involved an initial assessment, an educational intervention, and 1-month and 12-month follow-ups. Of those, 563 declined and 520 completed the baseline questionnaire (427 physicians and 93 mid-level providers). Each participant was asked to identify a “spiritual care coordinator” (SPC) in their practice (a nurse, medical assistant, or other staff member) to assist in providing spiritual care by offering additional resources to patients who indicate a need for further spiritual support in addition to what the physician provides. SPCs were also asked to complete the survey (*n* = 217). Final sample size was 737 (at least three-quarters of whom had received little or no training from AHS on integrating spirituality into patient care). The study was approved by the Institutional Review Boards of Duke University (Pro00054912) and AHS.

### Questionnaire

The 38-item questionnaire developed by study investigators assessed demographics, practice characteristics, personal religious characteristics, attitudes toward taking a SSH, and present and anticipated future behaviors concerning the SSH. Demographics included age, gender, race/ethnicity, and profession (physician, mid-level provider, nurse, social worker, etc.). Practice characteristics assessed were hospital of primary affiliation, medical specialty, years in practice, practice status, and years of employment. Religious involvement was measured by affiliation and importance of religion in daily life. Religious affiliations assessed were Christian, Jewish, Hindu, Muslim, Buddhist, other religion, and none. Christian participants were asked to specify a denomination. Religious importance was assessed by the statement “How important is religion in your daily life?” with responses on a 6-point scale from “not at all” (1) to “very much” (6); this item, like all others, was created by study authors.

The SSH was described for participants as follows: "By 'screening' spiritual history, this means taking 2-3 minutes to ask questions about the patient’s religious or spiritual (R/S) background if any, whether faith gives them hope, whether they have any spiritual beliefs that might influence medical decisions, or if other spiritual concerns are present that need to be addressed for health reasons. This is different from a chaplain’s spiritual assessment, which is a more comprehensive evaluation." The word “spiritual,” however, was not defined for participants.

#### Attitudes

Participants were asked: (a) “Do you think health professionals [HPs] should take the time to do a SSH?”(“not at all” [1] to “very much” [6]); (b) “If a SSH is taken, which HP should do it?” (physician, nurse/medical assistant, social worker, admission’s clerk, or other [specify]); and (c) “How many questions should a screening spiritual history consist of?”(1 to 6).

Participants were also asked: “On what kinds of patients should the SSH be taken?” Possible responses were (1) all outpatients, (2) outpatients during well-visit exams, (3) outpatients with chronic illness, (4) outpatients with terminal illness, (5) all inpatients when admitted to hospital, (6) inpatients with chronic illness, (7) inpatients with acute serious illness, (8) inpatients with terminal illness, and (9) other (specify). Following this question, participants were asked: “What should HP do with information from spiritual history if spiritual needs are present?” Response options were: (1) document in medical record, (2) be aware of, but not document, (3) refer to chaplain, (4) contact patient’s clergy, (5) contact social worker, and (6) address spiritual needs him/her self. “No” or “yes” answers were requested for each of the responses listed above; however, many participants responded by circling only the “yes” option because of the way responses were formatted. If “yes” was circled for any item in the block, then missing responses were designated as “no” (25% and 26%, respectively, for those questions).

Finally, HPs were asked: “In your experience, patients most often respond to a screening spiritual history with…” (a) resistance, (b) puzzlement, (c) indifference, (d) acceptance, or (e) appreciation.

#### Behaviors

Participants were asked: “How often do you currently take a SSH?” (“never” [1] to “always” [6]). Next, they were asked: “Would you be willing to take the time to administer a SSH?” (“no, never” [1] to “yes, very often” [6]). Third, participants were asked: “Would you be willing to take the time to review the results of a SSH?” (“no, never” [1] to “yes, very often” [6]).

### Statistical analyses

Analyses were stratified by professional group. Demographic, religious, and practice characteristics were described using frequencies and means, and were compared across the three groups using the chi-square statistic for categorical variables, Mantel-Haeszel chi-square or MHχ^2^ for ordinal variables, and analysis of variance for continuous variables. Bivariate associations between HP attitudes/behaviors and demographic, practice, and religious characteristics were first examined. Characteristics significant in bivariate analyses at *p* < 0.10 were included in regression models to identify independent predictors of attitudes/behaviors in each professional group. For dependent variables where responses ranged from 1 to 6, general linear regression was used based on theoretical grounds that justify the use of parametric statistics for ordinal data with five or more response categories [39, 40]. With regard to dependent variables with dichotomized responses categories (yes-no), logistic regression was used. Significance level for final results was set at alpha = 0.05 and was not adjusted for multiple comparisons given the exploratory nature of these analyses. The SAS statistical package (version 9.3; SAS Institute Inc., Cary, North Carolina) was used for all analyses.

## Results

Sample characteristics by professional group are presented in Table 1. The most common medical specialty among physicians was family medicine (34.3%), followed by surgery (16.0%), general internal medicine (12.2%), cardiology (5.6%), oncology (3.3%), pediatrics (1.6%), other internal medicine specialties (7.5%), and emergency medicine (0.5%). Physicians were older (45.4 ± 15.8 years) than MLPs (42.0 ± 13.7) and nurses/staff (39.0 ± 13.7), were more likely to be male (64.0% vs. 10.7% and 6.0%, respectively), were less likely to be white Caucasian than MLPs (56.1% vs. 81.3%), and were longer in practice (16.8 years vs. 11.6 years for MLPs and 10.2 years for nurses/staff).

**Table 1:** Characteristics of the sample (*n* = 737)

	Physicians	Mid-level Practitioners	Nurses, Other Health Professionals & Staff
	(*n* = 427)	(*n* = 93)	(*n* = 217)
	% (n) / Mean (SD)	% (n) / Mean (SD)	% (n) / Mean (SD)
Age, years	45.4 (15.8)	42.0 (13.7)	39.0 (13.7) ****
Gender (% female)	36.0 (151)	89.3 (83)	94.0 (204) ****
Race (% white)	56.1 (238)	81.3 (74)	57.1 (124) ****
Site (% Florida Hospital)	66.8 (279)	63.0 (58)	70.0 (149)
Specialty (% family med)	34.3 (146)	36.6 (34)	32.4 (70)
Years in practice	16.8 (11.3)	11.6 (9.5)	10.2 (10.2) ****
Employed/contract with AH, %	60.6 (258)	57.0 (53)	71.4 (152) *
If AH employ, years at AH	6.2 (7.4)	5.6 (6.8)	6.2 (6.7)
Religious affiliation, %			
Christian	77.9 (324)	91.1 (82)	94.2 (196) ****
Non-Christian	20.2 (84)	2.2 (2)	0.5 (1)
None	1.9 (8)	6.7 (6)	5.3 (11)
Christian denomination, %			
Catholic/Orthodox	23.3 (77)	25.3 (21)	17.9 (36) **
Protestant	33.0 (109)	34.9 (29)	42.8 (86)
Seventh-day Adventist	14.9 (49)	10.8 (9)	4.0 (8)
Other or missing	28.8 (95)	28.9 (24)	35.3 (71)
Seventh-day Adventist,			
% of total group	11.5 (49)	9.7 (9)	3.7 (8) **
Importance of religion, %			
Not at all/slight/some	16.4 (70)	15.1 (14)	15.7 (34)
Moderate	12.4 (53)	14.0 (13)	12.5 (27)
Quite a bit/very much	71.1 (303)	71.0 (66)	71.8 (155)

N may vary by <1% except where noted

*p < 0.05, **p < 0.01, ***p < 0.001, ****p < 0.0001 (by chi-square or analysis of variance)

AH = Adventist Health Services

Physicians were less likely to be Christian (77.9% vs. 91.1% for MLPs and 94.2% for nurses/staff). Among Christians, physicians were somewhat less likely to be Protestant but more likely to be Seventh-day Adventist (SDA) than nurses/staff, but were similar in denomination to MLPs. SDAs made up only a small minority of each professional group who were Christian, ranging from 4.0% of nurses/staff to 14.9% of physicians (11.5% of all physicians). Concerning importance of religion, no significant difference was found between the three groups; just over 70% indicated religion was important in daily life (“quite a bit” or “very much”).

### Attitudes and behaviors

#### Attitudes

Physicians were less likely than MLPs or nurses/staff to indicate that HPs should take a SSH (45.2% indicating quite a bit or very much vs. 52.7% of MLPs and 55.4% of nurses/staff, MHχ^2^ = 8.4, df = 1, *p* = 0.004) (Table 2). Concerning who should take the SSH, nurses/staff were more likely than physicians or MLPs to indicate *the physician* (55.1% vs. 41.8% of physicians and 29.4% of MLPs, χ^2^ = 30.1, df = 4, *p* < 0.0001). Physicians were more likely to indicate the SSH should be only 1–2 questions (27.5%) than did MLPs (22.6%) or nurses/staff (14.3%) (MHχ^2^ = 11.3, df = 1, *p* = 0.0005). The majority of all HPs agreed that three questions were ideal (50–56%). The majority of physicians and MLPs also agreed that patients respond with acceptance or appreciation (72.8% of physicians and 68.9% of MLPs vs. 55.8% of nurses/staff, χ^2^ = 18.9, df = 4, *p* = 0.0008), as compared to resistance, puzzlement, or indifference (27.2% of physicians, 31.1% of MLPs, 44.2% of nurses/staff).

**Table 2:** Attitudes concerning the “screening” spiritual history

	Physicians	Mid-level Practitioners	Nurses, Other Health Professionals & Staff
	(*n* = 427)	(*n* = 93)	(*n* = 217)
	% (n)	% (n)	% (n)
Should health professionals do a screening spiritual history (SH)?^2^			
Not at all/slight/some	27.7 (117)	22.6 (21)	18.3 (39) **
Moderate	27.2 (115)	24.7 (23)	26.3 (56)
Quite a bit/very much	45.2 (191)	52.7 (49)	55.4 (118)
Which health professional should do it?^3^			
Physician	41.8 (175)	29.4 (27)	55.1 (118) ****
Nurse or med assistant	33.9 (142)	31.5 (29)	17.8 (38)
Other (staff)	24.3 (102)	39.1 (36)	27.1 (58)
How many questions should screening SH consist of? ^2^			
One or two	27.5 (115)	22.6 (21)	14.30 (31) ***
Three	49.9 (209)	49.5 (46)	56.2 (122)
Four, five or six	22.7 (95)	28.0 (26)	29.5 (64)
Patients most often respond to screening SH with: ^2^			
Resistance	1.7 (7)	2.2 (2)	4.4 (9) ****
Puzzlement/indifference	25.5 (103)	28.9 (26)	39.8 (82)
Acceptance/appreciation	72.8 (294)	68.9 (62)	55.8 (115)
On what kinds of patients should SH be done? (% yes)^1, 3^			
All outpatients (OP)	46.0 (193)	52.8 (47)	59.0 (125) **
OP during well-visit	57.6 (240)	69.7 (62)	50.0 (106) **
OP with chronic illness	76.0 (317)	73.0 (65)	75.0 (159)
OP with terminal illness	80.1 (334)	82.0 (73)	82.6 (175)
All inpatients (IP) admitted	66.0 (275)	71.9 (64)	67.0 (142)
IP with chronic illness	71.9 (300)	74.2 (66)	72.6 (154)
IP with acute serious illness	72.9 (304)	75.3 (67)	74.5 (158)
IP with terminal illness	79.1 (330)	79.8 (71)	79.7 (169)
What should health professional do with spiritual history? (% yes)^1, 3^			
Document in medical record	69.3 (293)	76.3 (71)	68.2 (146)
Be aware of, not document	23.4 (99)	17.2 (16)	22.4 (48)
Refer to chaplain	82.3 (349)	90.3 (84)	91.2 (196) **
Contact patient’s clergy	35.7 (151)	39.8 (37)	30.8 (66)
Contact social worker	39.4 (167)	46.2 (43)	30.2 (65)*
Address spiritual needs	50.4 (213)	44.1 (41)	41.1 (88)

^1^Assuming that response to items not indicating “yes” were “no”

^2^Mantel-Haenszel χ^2^; ^3^chi-square = χ^2^

*p < 0.05, **p < 0.01, ***p < 0.001, ****p < 0.001

Patients most appropriate for a SSH were outpatients with terminal illness (80.1–82.6%), inpatients with terminal illness (79.1–79.8%), outpatients with chronic illness (73.0-76.0%), and inpatients with acute or chronic illness (71.9–76.0%). Physicians were less likely than MLPs or nurses/staff to indicate the SSH was appropriate for all outpatients (46.0% vs. 52.8% and 59.0%, respectively). With regard to what should be done if spiritual needs were present, the majority said this should be documented in the medical record (68.2–76.3%) and referral to a chaplain made (82.3–91.2%). Interestingly, 50% of physicians indicated they would address patients’ spiritual needs themselves.

#### Behaviors

The majority of physicians (55.1%), MLP (55.0%), and especially nurses/staff (70.3%) indicated they never or only rarely take a SSH (MHχ^2^ = 7.0, df = 1, *p* = 0.008); 17.0% of physicians said they often or always did so, compared to 15.4% of MLPs and 11.5% of nurses/staff (Table 3). With regard to future behavior, most HPs were willing to take a spiritual history (85.1% of physicians, 93.6% MLPs, 86.7% nurses/staff) or review the results thereof (86.9% physicians, 96.5% MLPs, and 85.6% nurses/staff). Few (1% or less) were unwilling to either perform or review a SSH.

**Table 3:** Present and future behaviors concerning “screening” spiritual history

	Physicians	Mid-level Practitioners	Nurses, Other Health Professionals & Staff
	(*n* = 427)	(*n* = 93)	(*n* = 217)
	*% (n)*	*% (n)*	*% (n)*
How often do you currently take a screening spiritual history?			
Never or rarely	55.1 (233)	55.0 (50)	70.3 (147) **
Sometimes	27.9 (118)	29.7 (27)	18.2 (38)
Often	10.4 (44)	8.8 (8)	4.8 (10)
Very often or always	6.6 (28)	6.6 (6)	6.7 (14)
Willing to do a spiritual history?			
No, never	0.7 (3)	1.1 (1)	0.5 (1)
No, but not opposed	9.2 (39)	4.3 (4)	9.0 (19)
No, could be convinced	5.0 (21)	1.1 (1)	3.8 (8)
Yes, sometimes	51.0 (215)	45.2 (42)	48.8 (103)
Yes, often or very often	34.1 (144)	48.4 (45)	37.9 (80)
Willing to review results of the spiritual history?			
No, never	0.6 (2)	1.1 (1)	0.5 (1)
No, but not opposed	8.0 (28)	2.3 (2)	8.2 (16)
No, could be convinced	4.6 (16)	0.0 (0)	5.6 (11)
Yes, sometimes	45.6 (159)	44.2 (38)	44.6 (87)
Yes, often or very often	41.3 (144)	52.3 (45)	41.0 (80)

**p < 0.01 (chi-square)

### Correlates of attitudes and behaviors

#### Physicians

With regard to attitude toward taking a SSH, bivariate analyses (not shown) indicated that family physicians, those more religious, and Christians (vs. non-Christian) were more likely to support this practice. Multivariate analyses indicated that personal religious characteristics (Christian affiliation and importance of religiosity) were the primary predictors (Table 4).

**Table 4:** Predictors of attitudes and behaviors concerning the “screening” spiritual history

	Physicians	Mid-level Practitioners	Nurses, Other Health Professionals & Staff
	*B(SE)*	*B(SE)*	*B(SE)*
Health professionals should do a screening spiritual history (range 1–6)			
Age	----	0.021 (0.009)*	----
Race	----	----	−0.13 (0.16)
AHS employee/contractor	----	−0.27 (0.24)	----
Specialty (family medicine)	0.09 (0.12)	----	----
Non-Christian affiliation	−0.30 (0.14)*	----	----
No religious affiliation	----	0.28 (0.57)	−0.34 (0.39)
Importance of religion	0.41 (0.04)****	0.53 (0.11)****	0.49 (0.06)****
*Model R-square (n)*	0.22 (423)****	0.36 (*n* = 93)****	0.29 (213)****
Physician is health professional who should do spiritual history (yes = 44% overall)			
Race	0.38 (0.22)	----	----
AHS employee/contractor	----	−0.81 (0.48)	----
Site (Florida Hospital)	−0.60 (0.22)**	−0.67 (0.47)	----
Specialty (family medicine)	0.53 (0.22)*	----	----
No religious affiliation	−0.97 (1.15)	----	----
Importance of religion	0.36 (0.09)****	----	----
*Likelihood ratio (n)*	41.4 (407)****	5.37 (91)	----
Patients most often respond to spiritual history with acceptance/appreciation (yes = 66%)			
Age	----	0.045 (0.023)	----
Years in practice	----	0.01 (0.04)	----
AHS employee/contractor	----	−1.30 (0.57)*	----
Specialty (family medicine)	0.33 (0.25)	----	----
Non-Christian affiliation	−0.27 (0.27)	----	----
Importance of religion	0.13 (0.08)	0.36 (0.20)	0.21 (0.11)
*Likelihood ratio (n)*	7.00 (404)	19.3 (90)***	3.61 (205)
All outpatients should receive spiritual history (yes = 50%)			
Age	----	0.043 (0.022)	----
Gender	−0.49 (0.23)*	----	----
Race	−0.40 (0.22)	----	----
Site (Florida Hospital)	0.71 (0.23)**	----	----
AHS employee/contractor	−0.19 (0.22)	----	----
Years in practice	----	0.055 (0.034)	----
Specialty (family medicine)	−0.51 (0.24)*	----	----
Importance of religion	0.29 (0.09)***	0.29 (0.19)	0.27 (0.11)*
*Likelihood ratio (n)*	42.9 (401)****	18.5 (89)***	6.7 (211)**
Spiritual history should be documented in medical record (yes = 70%)			
Gender	0.26 (0.24)	0.62 (0.81)	1.69 (0.62)**
Race	----	−1.85 (1.07)	----
Specialty (family medicine)	0.69 (0.25)**	1.14 (0.62)	----
*Likelihood ratio (n)*	11.5 (415)**	8.9 (91)*	8.2 (214)**
Current frequency of taking a spiritual history (range 1–6)			
Age	0.009 (0.004)*	0.015 (0.010)	0.007 (0.007)
Gender	0.21(0.12)	----	----
Race	−0.31 (0.12)*	----	----
Site (Florida Hospital)	−0.17 (0.12)	----	----
Specialty (family medicine)	0.37 (0.12)**	----	----
Years in practice	----	0.024 (0.014)	0.016 (0.009)
AHS employee/contractor	−0.30 (0.12)*	----	----
Non-Christian affiliation	−0.31 (0.14)*	----	----
Importance of religion	0.19 (0.04)****	0.10 (0.08)	0.19 (0.06)**
*Model R-square (n)*	0.16 (404)****	0.14 (91)**	0.08 (208)***
Willing to do a spiritual history (range 1–6)			
Age	0.003 (0.004)	0.016 (0.007)*	----
Gender	----	0.53 (0.30)*	----
Site (Florida Hospital)	----	----	0.21 (0.16)
Years in practice	0.006 (0.006)	----	----
Non-Christian affiliation	−0.07 (0.12)	----	----
No religious affiliation	----	−0.11 (0.45)	----
Importance of religion	0.21 (0.04)****	0.27 (0.08)**	0.23 (0.06)****
*Model R-square (n)*	0.10 (422)****	0.25 (93)****	0.08 (207)***

*p < 0.05, **p < 0.01, ***p < 0.001, ****p < 0.0001, "----" not significant in bivariate analyses

B = unstandardized beta, SE = standard error (from general linear model for continuous outcomes or from logistic regression model for dichotomous outcomes)

Concerning who should take the SSH, those most likely to indicate *the physician* were white, family medicine, and religious physicians. In contrast, physicians at Florida Hospital (AHS’ flagship hospital) and those with no religious affiliation were less likely to indicate this. Multivariate analyses revealed that family medicine specialty, religiosity, and non-affiliation with Florida Hospital were independent predictors.

With regard to patients’ responses, family physicians and highly religious physicians were more likely to indicate acceptance or appreciation; non-Christians were less likely to do so. No characteristics, however, were significant in multivariate analyses.

Concerning appropriate patients to receive the SSH, with “all outpatients” being the outcome, Florida Hospital-associated and highly religious physicians were more likely to indicate this, whereas male, white, AHS employee, and family physicians were less likely. Multivariate analyses revealed that female gender, Florida Hospital-affiliated, non-family physicians, and religiosity were predictive of screening all outpatients.

With regard to documenting the SSH in the medical record (MR), the only bivariate correlates were female gender and physician specialty, and multivariate analyses indicated only family medicine specialty predicted documentation.

Concerning present behavior, those more likely to take a SSH were older, female, non-white, non-Florida Hospital affiliated, non-AHS employed, family medicine, highly religious, and Christian physicians. Independent predictors were older age, non-white race, family medicine specialty, non-AHS employed, Christian affiliation, and especially, high importance of religion.

Willingness to conduct a SSH in the future was more likely among older physicians, those with more years in practice, Christian affiliated, and those with greater religiosity. Once religiosity was controlled, however, all other predictors lost significance.

#### Mid-level providers

Many characteristics predicting positive attitudes/practices in physicians were similar in MLPs. Concerning attitude towards conducting a SSH, MLPs more likely to endorse this practice were older, non-AHS employees, Christian (vs. none), and highly religious. In multivariate analyses, however, only religiosity and older age were predictors. With regard to agreement that physicians should do the SSH, non-AHS employed and non-Florida Hospital affiliated MLPs were more likely to feel this way. However, neither of these was predictive in multivariate analyses.

Patient acceptance or appreciation of the SSH was more likely reported by older MLPs, those longer in practice, non-AHS employed, and the more religious. Only non-AHS employed, however, retained significance in multivariate analyses. Predictors of all outpatients receiving the SSH were older age, longer in practice, and greater religiosity. None were predictive in multivariate analyses, although older age came close (*p* = 0.054). Concerning MR documentation, MLPs favoring this practice were more likely to be female, non-white, and family medicine affiliated, although none of these was predictive in multivariate analyses.

Regarding current behavior, older MLPs, those longer in practice, and the more religious were more likely to take a SSH, although no characteristic reached significance in multivariate analyses. Willingness to take a SSH was predicted by older age, female gender, greater religiosity, and Christian affiliation (vs. none). All except affiliation maintained statistical significance in multivariate analyses.

#### Nurses/staff

Compared to physicians and MLPs, fewer characteristics predicted nurses/staff attitudes/practices. Only religiosity was an independent predictor of positive attitudes toward taking SSH, patient acceptance or appreciation, screening all outpatients, and willingness to take the SSH. No specific characteristics predicted feeling *the physician* should take SSH, and being female was the only characteristic that predicted MR documentation. Current taking of the SSH was more common in those older, longer in practice, and more religious, although only religiosity was predictive in multivariate analyses.

## Discussion

To our knowledge, this is the first study of a faith-based health system to examine attitudes/practices of HPs toward taking a screening spiritual history in an outpatient setting. Although faith-based, the AHS does not require or give preference to hiring Christians or Adventists, nor does it preferentially care for Christian or Adventist patients (although some Adventist practitioners and patients may understandably gravitate towards this health system). Thus, characteristics of participants and their attitudes/practices were not that different than those reported in other studies of physicians and MLPs. Physicians in our study were somewhat more likely to be Christian (78%) compared to physicians in the largest nationwide survey of physicians to date (61%) [24], but were similar to Americans more generally (71%) [41]. The present sample also had more Adventist physicians (11.5%) compared to American Adventists in general (0.5%).

Other characteristics of physicians were similar to those in the Curlin et al. nationwide study [24]. Average physician age in the latter was 49 (vs. 45 here); 22% were Catholic (vs. 23%); 39% were Protestant (vs. 33%); and 63% were moderate or high on religiosity (vs. 71% quite a bit or very much). Physicians in that study, though, were less likely to be women (26% vs. 36%), more likely to be white (78% vs. 56%), and less likely to be family physicians (14% vs. 34%). They also had similar SSH attitudes to our physicians, with 55% indicating it was usually or always appropriate to inquire about R/S (vs. 27% indicating moderate and 45% highly favorable attitudes here). Likewise, Curlin and colleagues found that 10% of physicians often or always inquired about patients’ R/S issues, compared to 17% of our physicians. A systematic review involving 20,000 physicians from 34 studies found that 34% (median) often or always took a SSH (although that review included psychiatrists, where median was 50%) [31]. Thus, our finding of 17% is in line with other studies of general physicians.

With regard to MLPs, systematic data on SSH attitudes/ behaviors are sparse [42] despite insistence by the National Organization of Nurse Practitioner Faculties that spiritual care be included in educational curricula (with identified spiritual competencies required) [43]. In a 2001 report on 102 Indiana NPs, Stranahan reported 24% often or always talked with patients about R/S (vs. 17% of MLPs here), and 30% indicated they rarely or never did (vs. 53% here) [44]. These findings are consistent with a 2006 study of 65 NPs in North Carolina where 61% rarely or only occasionally talked with patients about R/S matters [45]. While there is considerable interest in addressing spiritual issues by physician assistant (PA) training programs [46], we found only one study examining SSH attitudes/practices. Assessed were 334 of 779 licensed PAs in Kansas. Of those, 89% agreed that PAs should be aware of the patient's R/S, 70% said they should inquire about patients’ R/S beliefs, and 61% indicated they at least “sometimes” inquired about patients’ R/S beliefs (although <10% frequently or always did so) [47]. Again, PA religiosity was the strongest correlate of positive attitudes and behaviors.

Concerning nurses/staff more generally, professional healthcare associations worldwide acknowledge in their standards of practice that nurses should address spiritual issues (see Sessanna et al. for review) [48]. Many studies have explored nurses’ perceptions of spiritual care [49–51] and the findings of these studies indicate that nurses believe that spiritual care is a key aspect of the nursing role, although they lack of education and training on the delivery of spiritual care. With regard to taking a spiritual history, though, there is almost no systematic research in this regard [52]. Despite an exhaustive review of the literature, we could locate only one study examining how often nurses take a SSH (and that focused on hospital nurses). Researchers surveyed 120 acute care nurses from different services at New York Presbyterian [53]. Nearly 80% answered yes to the question, “Do you currently screen for spiritual needs?” and 96% said addressing patients’ spiritual needs was within their professional role. However, 50% also believed that “a patient’s spirituality is private to the individual and should not be invaded upon by the nurse.” What exactly was meant by “spirituality” or “spiritual needs” was unclear. Since there is no general agreement on those terms, it could mean an assessment of purely psychological or emotional issues [54], not anything distinctively spiritual, religious, or related to the transcendent dimensions of illness that sick patients (a) struggle with, (b) need expert pastoral care to work through, or (c) affect medical decisions, quality of life, or medical outcomes [55].

Furthermore, if patients’ spiritual concerns relate to broader psychological issues, they may benefit from having these concerns addressed by a psychologist, counselor, or other expert, rather than pastoral care specialists.

Nurses/staff were more likely to identify the physician as the best person to take a screening spiritual history. This is a notable finding, perhaps reflecting less clinical experience of nurses/staff in this regard compared to physicians or MLPs, or that nurses/staff felt they had less clinical autonomy to do so than these other clinicians. Furthermore, half of physicians said they would address spiritual needs of patients themselves and fewer physicians than either MLPs or nurses/staff felt that patients should be referred to a chaplain to have spiritual needs met. Interestingly, nurses/staff seemed to intuitively realize that while physicians as head of the healthcare team should be taking the SSH, they did not have the time or the expertise to address the spiritual needs uncovered by the SSH (which we would agree with).

Our findings on *predictors* of positive attitudes toward the SSH and actual behaviors in this regard are consistent with those in the literature. In Curlin et al.’s nationwide study, R/S inquiry was strongly predicted by physician R/S [24]. Of those with low personal R/S, only 23% “ever” inquired about patients’ R/S compared to 76% of physicians with high personal R/S. Those feeling uncomfortable with the topic were least likely to inquire (OR = 0.60, 95% CI 0.40–0.90), consistent with a recent meta-analysis of physician inquiry finding that physician R/S and training were primary in predicting whether a physician took a SSH [31]. Of course feeling comfortable with the topic does not always mean that a clinician is competent in addressing it. Other predictors of physician inquiry in the Curlin et al. study were female gender, Christian religion, and primary care specialty, similar to characteristics identified here. Barriers to physician inquiry were lack of time, personal discomfort, colleagues’ or institutional disapproval, and concern over different belief systems, similar to concerns voiced by oncologists and oncology nurses [38]. Training is essential because the way an HP takes a SSH will determine whether patients open up on this private and personal topic [56].

Unique to our study, though, were inquiries about which professional should do the SSH, number of questions that should be asked, patients’ responses to the SSH, what kinds of patients should receive the SSH, what HPs should do with the information, willingness to review a SSH taken by others, and identification of predictors of these attitudes, which to our knowledge have not been reported in the literature.

### Limitations

First, participants in this study were a convenience sample of HPs, particularly those with an interest in the topic and largely from the Southeastern U.S. Thus, the findings here represent a “best case scenario” with regard to attitudes and behaviors. Second, responses to some questions may have been influenced by formatting issues (see Methods). Third, the reliability of responses to this single administration of the questionnaire is unknown. However, the large sample, the geographical diversity involving HPs from several different states, and being one of the first reports from a large FBHS make this study unique. Fourth, we did not correct our *p* values for multiple comparisons, given the exploratory nature of this study, increasing the possibility of significant associations by chance alone. Finally, we did not define the word “spiritual” for participants or identify participants who were spiritual but not religious. Thus, physicians (who were less religious than others in this study) may have been less likely to say they would do a spiritual screening because they thought it was only about religion and not about spirituality as defined more broadly than religion. Perhaps if we had provided a definition for spirituality and specified that it could be anything the patient wanted it to mean, physicians (and others) might have been more likely to do a screening.

## Conclusions

This is the first study to examine attitudes/behaviors regarding the SSH among outpatient clinicians and staff in a large faith-based health system, to compare those attitudes/behaviors between each clinician type, and to identify predictors of attitudes/behaviors in each provider type. Even in AHS with an explicit mission to provide spiritual care, only a small minority of physicians, MLPs, and nurses/staff often or always take a screening spiritual history (11% to 17%). Many, however, indicate that a SSH should be done (45% to 55%) and are willing to do it (85% to 94%). Between one-third and one-half believe that *the physician* should do it, especially physicians and nurses/staff. HPs who are older, female, non-white, Christian, more religious, more years in practice, or in family medicine settings have more positive attitudes toward the SSH and are more likely to conduct one. For now, the clinical implications of this research study (and the broader literature) are that (1) a brief SSH should be taken on patients with chronic illness or those struggling with emotional issues (i.e., any patient that is having difficulty coping with illness), (2) the physician or MLP should take the SSH, and (3) clinic staff need to know how to assist the clinician in addressing spiritual needs when they are identified. More importantly, training is needed for each of these provider types on how to take a spiritual history and what to do with the information learned. Whether an intensive training program on how to integrate spirituality into patient care will affect these attitudes/practices is the next important step in this research agenda.
